# Are the Italian Children Exposed to Advertisements of Nutritionally Appropriate Foods?

**DOI:** 10.3390/foods9111632

**Published:** 2020-11-08

**Authors:** Daniele Nucci, Filippo Rabica, Giulia Dallagiacoma, Cristina Fatigoni, Vincenza Gianfredi

**Affiliations:** 1 Digestive Endoscopy Unit, Veneto Institute of Oncology IOV-IRCCS, Via Gattamelata 64, 35128 Padua, Italy; daniele.nucci@iov.veneto.it; 2 Department of Pharmaceutical Science, University of Perugia, Via del Giochetto 2, 06123 Perugia, Italy; filippo.rabica@libero.it (F.R.); cristina.fatigoni@unipg.it (C.F.); 3 Post-Graduate School of Hygiene and Preventive Medicine, Department of Public Health, Experimental and Forensic Medicine, University of Pavia, 27100 Pavia, Italy; giulia.dallagiacoma01@universitadipavia.it; 4 Post-Graduate School of Hygiene and Preventive Medicine, Department of Experimental Medicine, University of Perugia, P.le L. Severi 1, 06122 Perugia, Italy; 5 School of Medicine, Vita-Salute San Raffaele University, 20132 Milan, Italy; 6 CAPHRI Care and Public Health Research Institute, Maastricht University, 6200 MD Maastricht, The Netherlands

**Keywords:** television, marketing, advertising, children, Italy, food, nutritive value

## Abstract

Unhealthy eating habits are one of the main risk factors for overweight/obesity, and food marketing plays a major role in their development. The aim of this study was to monitor the amount and the characteristics of food marketing directed to Italian children broadcasted on television (TV). The WHO tool to assess food and beverage multimedia marketing aimed at children was used to analyze TV recordings. Type of product branded, viewing time, channel’s target, and broadcasting company were the exposure variables analyzed. The power of persuasive techniques was also assessed. Food products were categorized as either core or non-core products on the basis of their nutritional profile. A total of 320 h of TV broadcasting was analyzed, including 51.7 h of commercials. Food and beverages were the second most frequently advertised products, with an average of 6 food advertisements per hour during peak viewing time. A total of 23.8% of food advertisements were recorded during the time slot of 3:00 p.m. Considering food and beverage commercials, “humor” was the most frequently used primary persuasive technique, while the “image of the product/packaging” was the most commonly used secondary persuasive technique. Products specifically targeted to children were 94.3% non-core. Our findings indicate that core foods are highly underrepresented in TV commercials, especially during children TV programs and peak viewing time.

## 1. Introduction

According to the World Health Organization (WHO), 40 million young children aged under 5 and 131 million of children aged 5–9 are either overweight or obese [1,2]. This alarming phenomenon is stably increasing over time, mainly in high-income countries. In Europe, Italy has the highest prevalence of overweight and obesity in children aged 6–9 years (boys: 21% overweight and 9% are obese; girls: 38% overweight and 14% obese) [3,4]. The spread of unhealthy dietary patterns, mainly based on processed food (PF) and ultra-processed food (UPF) consumption, is one of the leading causes of this phenomenon. Several pieces of evidence have shown a strong association between the consumption of these types of foods and an increased risk of excess weight, overweight, and obesity [1,5]. Indeed, although genetics can play a role in the onset of overweight and obesity [6,7], environmental factors and lifestyle remain the leading causes of weight gain [8,9]. Modern cities are full of the so-called “obesogenic environment” risk factors, mainly characterized by low chances of practicing physical activity and easy access to fast food and highly processed foods, leading to an intake of food that is high in calories and poor in nutrients with a reduced energy expenditure [10].

Sedentary lifestyle and unhealthy food choices are crucial factors in the etiopathogenesis of overweight and obesity. Studies have found that the number of hours of screen time can have a great impact on children’s behavior, especially the hours spent watching television (TV) [11,12,13]. It was highlighted that watching TV, as well as screen time with any other device, for longer than what the recommendations suggest (1–2 h/day), can lead to an increased risk of overweight and obesity [14,15,16]. There are two potential mechanisms behind this: first, watching television is a leisure activity, and an increased time spent in front of the TV reduces energy expenditure [17]; secondly, TV is a medium to convey unhealthy eating behaviors through food marketing, to which children are massively exposed [18]. Food marketing is not only responsible for conveying unhealthy eating behaviors, it was also proven to increase the daily intake of food and calories in children [19], leading to the spread of childhood obesity and increasing the risk of obesity-related non-communicable diseases at a young age [20]. Previous evidence in food marketing showed that (ultra)-processed foods (for instance, sugary cereals, soft drinks, salty snacks, candies, and fast food meals) represent 60–90% of all food categories advertised worldwide [21]. In light of these considerations, it is crucial to monitor food marketing targeted to children.

The aim of this study was to monitor the amount of food marketing directed to Italian children broadcasted on TV. For this purpose, both the exposure to food advertising and the techniques used by food companies to persuade children to consume their products were measured.

## 2. Material and Methods

### 2.1. Sampling

We used the protocol study developed by the WHO that aims to monitor children’s exposure to food marketing [22]. In order to identify the 5 most watched TV channels by children aged 1–16, we developed an ad hoc questionnaire and administered it to a sample of 33 Italian parents who were voluntarily recruited through advertisements. The anonymous questionnaire was based on 4 items aimed at investigating the number of children, children’s gender, and age, as well as the 5 most watched TV channels for each child. The age classes were categorized into 6 groups as follows: 1–5 years; 6–10 years; 11–13 years; 14–16 years; 17 years; and lastly, 18 years old. The 4-item questionnaire was based on multiple choice questions; however, the list of channels was accompanied by a free-text box, allowing parents to add further channels viewed by their children (Table S1). The questionnaire was developed by a research member (F.R.), and the research team assessed the intelligibility and clarity of the questions (D.N., V.G., G.D., C.F.). The 5 most watched TV channels resulting from the questionnaire were recorded from 6:00 a.m. until 10:00 p.m. for 20 non-consecutive days (2 weekdays and 2 weekend days), in the period July to November 2018, excluding holiday breaks. After the video acquisition, all the recorded material was stored and scanned, searching for advertisements.

### 2.2. Commercial Coding

All commercials broadcasted by the selected TV channels were evaluated through a tool developed by the WHO to assess food and beverage multimedia marketing addressed to children [22]. Items that were part of a program in the opening and closing credits, sponsorships, and promotions for content to appear later were not considered as advertisements.

All the analyzed parameters were grouped into 2 categories: the first one referred to the “exposure” and the other referred to the “power” of the persuasive techniques. In this context, the exposure was determined by the percentage of target people reached by the marketing message during a specific time period and by the frequency or the number of times people were exposed to the marketing message. Power of persuasive techniques refers to the creative content, design, and execution of the marketing message [23]. Some of the exposure variables (type of product, viewing time, channel’s target, and broadcasting company) were assessed for all types of commercials, while some specific exposure variables (duration of each food advertisement, type of program, and target of the product advertised) were only assessed for food and beverage commercials. Power of persuasive variables were assessed only for food and beverage marketing.

#### 2.2.1. Assessing Exposure Variables

Type of product branded, viewing time, channel’s target, and broadcasting company were assessed for each commercial. Specifically, products were categorized according to the 19 commercial categories ranging from food and beverage to household equipment or toys [22]. Moreover, since supermarkets commercials were not included in the WHO’s list, they were added as a supplementary category. Referring to the viewing time, the 16 h of TV programs recorded were divided into 32 time slots of 30 min each. Time slots were then categorized as “peak viewing time” and “non-peak viewing time” of children. Peak viewing time of children consists of the period of time during which the share of children watching television is greater than 25% [22]. According to previous studies, the time slot between 5:00 p.m. and 8:00 p.m. may be considered as peak viewing time for children [24,25]. The average number of commercials broadcasted in 1 hour of both peak viewing time and non-peak viewing time was assessed. The channel’s target was defined as “children” or “general public”, and lastly broadcasting companies were categorized into public and private companies. The channels were considered public if the channel belonged to the national free-to-air public broadcasting company of Italy (RAI—Radiotelevisione Italiana), and were considered private if the channel belonged to private free-to-air broadcasting companies.

Only for food and beverage advertisements, the duration of advertisement, type of program, day of the week, and target of the product advertised were assessed. The duration of each advertisement was assessed and expressed in seconds, estimating the mean duration of food commercials. The type of program (e.g., children’s show, drama, comedy; Table S2) during which food commercials were displayed, and the day of the week (weekdays or weekend days) were recorded. Lastly, the target of the products advertised was determined and categorized into (i) children, (ii) children and teenagers, (iii) teenagers, (iv) teenagers and adults, (v) adults, (vi) the elderly, and (vii) the whole family [22]. Since the aim of the study was to assess the exposure to food marketing of children aged 1–16 years old, we grouped the first three categories together in order to provide the exposure of the young public. The target of the commercials was determined on the basis of the message conveyed or the main characters of the commercial.

#### 2.2.2. Assessing the Power of Persuasive Techniques

Persuasive techniques were divided into two groups: (i) primary persuasive techniques and (ii) secondary persuasive techniques. Primary persuasive techniques were represented by the main message that commercials displayed to persuade customers into buying their products (accessible at https://euro.sharefile.com/share/view/se68de1782d74a8d8) [22]. The prevalence of each primary persuasive technique was independently assessed for all food and beverage commercials and food commercials targeted to a younger public. Secondary persuasive techniques were represented by additional parameters used in a commercial to increase the engagement to the products being advertised [22]. The secondary persuasive techniques evaluated were (i) image of the product/packaging, (ii) dynamic audio-visual components (e.g., use of lights or bright colors, sounds such as screams and loud noises), (iii) premium offers (i.e., prizes linked to product purchase), (iv) special characters (e.g., cartoon characters, licensed characters, child actors, and brand equity characters), (v) brand logo, and (vi) web address and social media logos. The brand logo was considered as a persuasive technique when it appeared on the screen as a separate image from the packaging of the product. Since several different secondary persuasive techniques could be used in a single commercial, each of them was separately taken into consideration [22].

### 2.3. Food and Beverage Products Coding

For each food and beverage product shown, we assessed the food category, core vs. non-core classification, and presence of added sugars. Each product was classified into a food category according to the WHO definition [22]. A further category for alcoholic beverages (such as wine, beer, and spirits) was added by the authors. Commercials that displayed meals and snacks consisting of more than one product were categorized as composite dishes. When more than 1 product appeared on the commercial, only the product that appeared for the most of the commercial’s time was considered. When more than 1 product received the same attention, all the products were considered. The prevalence of food and beverage in commercials specifically targeted to a young public was separately assessed.

All food and beverage products were classified into core and non-core products, depending on their nutritional profile. Products were considered “core” if they had a nutritional profile able to adequately provide the body with all the essential nutrients required, while “non-core” products were identified as those that were providing a surplus, in terms of nutrients or energy, to the nutritional requirements [26]. This classification of food into core and non-core was based on clearly defined cut-off points of fats and sugars determined by dietary guidelines [27,28]. According to the WHO nutrient profile model [29], core products may be allowed for advertisement to children, while non-core products should not be advertised to this age group. Composite dishes were considered as non-core when one or more of their components exceeded the limits. Lastly, the presence of any added sugars or non-sugar sweeteners was also evaluated.

### 2.4. Statistical Analysis

Descriptive characteristics of the advertisements recorded were presented as mean and standard deviation (SD) or percentages and numbers. To assess the differences between groups, we performed the chi-square test for categorical variables and Student’s *t*-test for continuous variables, as appropriate. For both tests, the degree of significance was set at *p* < 0.05. Statistical analysis of the data was performed with STATA SE 12.0 (StataCorp College Station, TX, USA).

## 3. Results

### 3.1. Children’s Most Watched TV Channels

A total of 33 parents agreed to fill the questionnaire, with a total of 33 responses and a sample of 57 children, mainly in the age group of 6–10 years (35%). The most watched channels were “Rai Yoyo” (12%), “Boing” (10%), “Rai Gulp” (10%), “Cartoonito” (10%), “Canale 5” (9%), and “Italia 1” (9%). Even though Rai Yoyo was the most viewed, this TV channel has been free from commercial advertisements since 2016, and thus it was not recorded for the advertisements assessment. However, this channel was considered when comparing data between public channels and private channels, as well as to compare channels targeted to the general public and channels targeted to children. Among the TV channels considered, four were targeted to children (Rai Yoyo, Rai Gulp, Boing, Cartoonito), while two channels were targeted to the general public (Canale 5, Italia 1). Furthermore, two out of six selected TV channels were public (Rai Yoyo, Rai Gulp) and four were private (Boing, Cartoonito, Canale 5, Italia 1).

### 3.2. Overall Marketing Exposure

During the 20 days of recording, 320 h of broadcasting TV were obtained, with a total of 9069 commercials identified, corresponding to a total of 51.7 h (16.2% of total programming time). Considering the whole sample of commercials, food and beverages were the second most frequently advertised products (19.4%), while the most frequent was toys (21.5%). On average, 27.7 commercials per hour were transmitted during non-peak viewing time, compared to 31 commercials per hour during peak viewing time. Channels targeted to the general public aired a higher number of commercials compared to channels targeted to children, with a borderline significant weak association (*t* = 2.045; *p* = 0.055). The private free-to-air channels transmitted a statistically higher number of total commercials compared to public free-to-air channels (*t* = 3.79, *p* = 0.010).

#### 3.2.1. Food and Beverage Marketing Exposure

As far as food and beverage commercials are concerned, a total of 1756 commercials were identified. An average of 5.4 food and beverage commercials per hour were displayed during non-peak viewing time, increasing to six during peak viewing time. Considering all time slots, the slot of 3:00 p.m. had the highest rate of food commercials (23.8% of the total of commercials displayed in this slot). Channels targeted to general public aired a statistically higher rate of food commercials compared to channels targeted to children (χ^2^ = 206.14; *p* < 0.001). Private free-to-air channels transmitted a higher number of food and beverage commercials compared to public free-to-air channels (χ^2^ = 27.97; *p* = 0.000).

**Figure 1:**
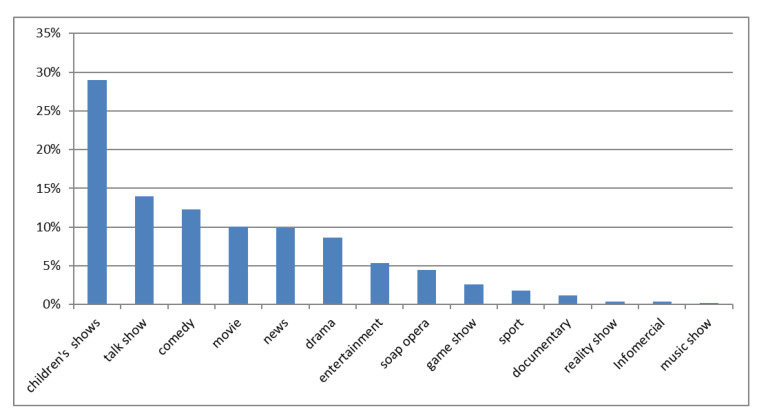
Distribution of food and beverage commercials among the types of programs. * Statistically significant (*p* = 0.000).

Food and beverage commercials covered 9.7 h out of 51.7 h of total commercials (18.8%). The length of food and beverage commercials ranged between 5 and 60 s, and most of the commercials had a length of 15 s (707 commercials). Children’s TV shows were the type of program during which food and beverage commercials were mostly broadcasted (29% of total commercials), compared to the rest of the shows scheduled (χ^2^ = 180.02; *p* < 0.001) (Figure 1). No significant differences were found between the total of food and beverage commercials aired during the weekdays (49%, *n* = 861/1756) compared to the total of food and beverage commercials aired on weekend days (51%, *n* = 895/1756).

#### 3.2.2. Food and Beverage Marketing Persuasive Techniques

**Figure 2:**
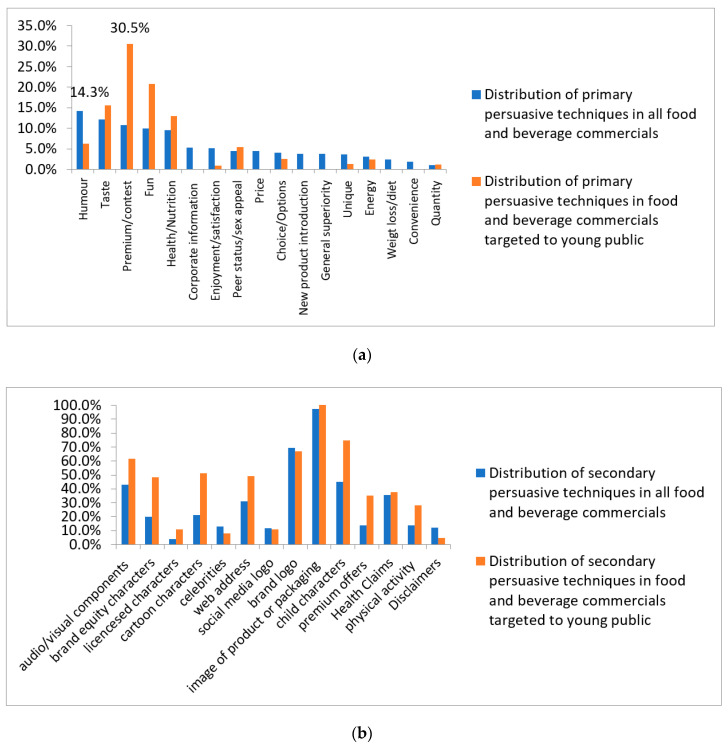
(**a**) Distribution of primary persuasive techniques and (**b**) of secondary persuasive techniques among food and beverage commercials.

Among primary persuasive techniques, “humor” was the most frequently used in all food and beverage commercials (14.3%) (Figure 2a). This technique focuses on jokes and sketches. Considering food and beverage commercials targeted to a young public, the most used primary persuasive technique was “premium/contest” that was used in 30.5% (*n* = 186/610) of food commercials targeted to the young public (Figure 2a).

As for secondary persuasive techniques, “image of the product/packaging” was the most often used secondary persuasive technique (97.3%) in all food and beverage commercials. The “brand logo” appeared in 69.4% of all food and beverage commercials (Figure 2b), and thus it was the second most used technique in all commercials. “Dynamic audio/visual components” were found in 43% of all food and beverage commercials. “Brand equity characters” were used in 20% of all food commercials, while “licensed characters” appeared in 3.9% of all commercials and “cartoon characters” were used in 21.3% of all food commercials. The “web address” of the brand appeared on screen in 31.3% of all commercials, and the “social media logo” was displayed in 11.9% of the commercials. A total of 13.9% of all commercials referred to “premium offers” that could be won by purchasing the product.

The most used secondary techniques in commercials targeted to a young public, apart from the image of the product itself (100% of food commercials targeted to young public), were the use of “children characters” in the commercials (74.6% of food commercials targeted to young public), and having the “brand logo” of the company appear on screen (66.9% of food commercials targeted to young public) (Figure 2b). On average, each food and beverage commercial targeted to a young public used six secondary persuasive techniques, while commercials addressed to the general public used on average 3.5 secondary persuasive techniques.

### 3.3. Food and Beverage Products in Television Marketing

Since more than one food/beverage could be advertised within the same commercial, a total of 1808 food and beverage products were identified among the 1756 food and beverage commercials, of which 610 were targeted to a young population (33.7%) (Table 1). Sweets, such as candies and desserts, were the most advertised category, both among all food and beverage products and among products targeted to the young public (22.1% and 30.5%, respectively) (Table 1). Composite dishes were the second most advertised products among all food and beverage commercials (17%) and among commercials targeted to the young public (28.9%) (Table 1). Products in this category range from fast food meals to frozen pizzas and burgers, as well as snacks made of more than one product (e.g., a snack composed of pie and orange juice). Food categories that should be the core of a healthy diet, such as fruits/vegetables and fish/meat, had a low prevalence (2.3% and 2.2%, respectively) among all food and beverage products. The low prevalence of fruits/vegetables and fish/meat dramatically decreased to a value of 0 when products targeted to children were analyzed (Table 1).

#### Core vs. Non-Core Foods and Beverages

Among all food and beverage products, 80.7% were non-core while 19.3% were core (Table 1). Products specifically targeted to children were 94.3% non-core and 5.7% core (Table 1). In each of the five channels recorded, the number of non-core products transmitted was statistically higher compared to core products (χ^2^ = 182.2617; *p* < 0.001).

**Figure 3:**
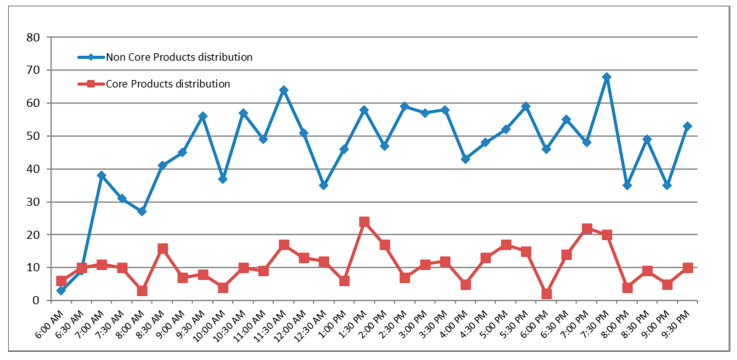
Distribution of core and non-core food among the time slots. Peak viewing time from 5:00 p.m. to 8:00 p.m.

The time slot of 7:30 p.m. (during peak viewing time of children) registered the highest number of non-core products (Figure 3). During the day, the transmission of core products was constantly lower than non-core products, with no statistical difference between the number of core products advertised during non-peak viewing time and core products advertised during peak viewing time. Moreover, in each time slot, the number of non-core products advertised was significantly higher than core products. On average, it was found that the broadcasting for non-core products was 4.4 products per hour during non-peak viewing time, which increased to 5.5 products per hour during peak viewing time. As for core products, the average broadcasting was of one product per hour during non-peak viewing time, that increased to 1.5 products per hour during peak viewing time.

Non-core products were statistically more frequently advertised using “image of the product/packaging” (χ^2^ = 87.14; *p* < 0.001), “brand logo” (χ^2^ = 3.90; *p* = 0.0048), “dynamic audio/visual components” (χ^2^ = 150.61; *p* < 0.001), “licensed characters” (χ^2^ = 6.48; *p* = 0.011), “cartoon characters” (χ^2^ = 7.16; *p* = 0.007), “social media logo” (χ^2^ = 8.12; *p* = 0.004), and “premium offers” (χ^2^ = 67.47; *p* < 0.001). No significant differences were found between non-core and core products for the use of “brand equity characters” (χ^2^ = 2.2407; *p* = 0.134) and the use of “web address” (χ^2^ = 0.75; *p* = 0.387).

## 4. Discussion

To the best of our knowledge, this is the first study monitoring TV advertisements targeted to Italian children. The study mainly focuses on quantity and quality evaluation of food and beverage advertisements, showing that Italian children are largely exposed to food and beverage advertisements that most of the time are classified as non-core foods. These results are in line with the most recent available data showing that in Italy, in 2016, the prevalence of children aged 6–10 years who spent more than two and five hours watching TV was 41.2% and 9.0%, respectively; moreover, 43.6% of children have a TV in their bedroom. Children who have TV in their room tend to watch it for more than 2 h a day. The presence of a TV in the bedroom is more frequently influenced by the parental education level [30]. Moreover, parental support is particularly necessary in order to navigate the complexities of the media and marketing environment, as well as to develop children’s ability to recognize the selling intent of advertisements [30,31]. Our study was conducted through recording the five Italian TV channels that are most watched by children, scanning them in search of commercials. Food advertisements were found to be the second most transmitted TV advertisement, representing 19.4% of all commercials. These findings are consistent with those in the study conducted by Kelly and colleagues, which showed that food advertisements were on average the second most transmitted commercial (18%) across the 11 countries considered [32]. Furthermore, consistent with previous studies, the current study found that private channels transmit a higher number of food advertisements compared to public channels [33].

Moreover, although food and beverage advertisements were predominantly broadcasted by channels targeted to the general public and mainly by private channels, the highest number of food and beverage commercials were transmitted during shows addressed to children. However, more than the prevalence of food advertisements, what really matters is the kind of food and beverages that are advertised. In the present study, we first analyzed food categories, and, consistently with previous studies [32,34], this showed that “sweets” were the most broadcasted food category, representing 22.1% of the advertised items in all food and beverage commercials, with 30.5% of advertisements specifically targeted to children; they were followed by composite dishes such as fast food meals (17% and 28.9% in all food and beverage commercials and commercials targeted to children, respectively), while fruits and vegetables were completely unrepresented. In addition, in our study, most of the analyzed food products in all commercials and those targeted to children were classified as non-core (80.7% and 94.3%, respectively) due to their poor nutritional quality. The results of the current study about the high rate of children exposure to non-core food advertisements are in line with other published findings highlighting that TV food advertisements usually focus on unhealthy products [35,36,37].

An interesting study conducted in Switzerland by Keller and colleagues showed how food and beverage commercials displayed in children’s TV programs convey a food pyramid with completely inverted layers compared to the recommended food pyramid, with sweets and fast food meals at the basis and fruit and vegetables at the top [38]. Consistent with these results, Cossenza-Quintana and colleagues [39] recently showed that 85% of the food advertisements aimed at children in Guatemala were non-core and should not be permitted if the WHO Nutrient Profile was followed. In this paper, in addition to the analysis of the nutritional profile of advertised foods (core vs. non-core products), the marketing strategies were also analyzed. The purpose of advertising is to persuade, and for that reason marketing strategies play a crucial role in driving consumer choices, with children being the most vulnerable members of the public in this context. Indeed, as confirmed by previous studies, children, especially those under 8 years of age, due to their cognitive development do not completely understand the persuasive message of the advertisements and thus are more susceptible to the effects of marketing compared to adults [40]. Noticeably, food commercials of non-core products make a greater use of persuasive techniques compared to commercials of core products, as previously reported by Vilaro et al. [41]. In 2014, Jenkin et al. performed an extensive systematic review and found that the most used persuasive techniques in food advertisements addressed to a young public were premium offers, promotional characters, nutrition or/and health-related claims, taste, and fun, mostly used to promote non-core food [42]. Consistently, in the current study, we found that the main persuasive technique used by food commercials targeted to a young public was related to premium offers/contest, with this being the main theme of 30.5% of commercials. Premium offers are used to persuade children to buy products with prizes that may be won either by purchasing the product or through a contest [43]. This result is similar to other studies, including one carried out in Australia that was based on a sample of 324 h of TV, where premium offers were the most common persuasive technique found in 39% of food advertisements [44]. Premium/contest and humor are persuasive techniques that can convey food and nutritional information through fun and enjoyment, effectively influencing food choices, especially amongst the youth [45]. This aspect should be considered, particularly when a media literacy-based intervention is planned. Previous studies have shown that media literacy-based intervention encouraged both parents and children to discuss advertisements while watching television together and while shopping at a grocery store in terms of when food choices are made [46]. Generally speaking, a good level of media literacy allows people to better understand the messages sent by media and to resist the persuasive media campaign, positively influencing their decision-making process [47].

The present study also shows that the recorded channels transmitted a daily average of 5.5 food commercials per hour and an average of six food commercials per hour during children peak viewing time. Moreover, regarding the amount of non-core products advertised, our results have shown that the channels analyzed transmitted an average of 4.4 non-core products per hour during non-peak time, which increased to an average of 5.5 non-core products per hour during children peak viewing time. Similar results were obtained by Kelly and colleagues [32]. In their multicenter study, Kelly et al. analyzed TV food advertising across different countries in Australia, Asia, Western Europe, and North and South America, showing an average of five TV food commercials per hour [32]. Lastly, it was possible to estimate the exposure to TV non-core products advertisements of Italian children—assuming that children watch TV during peak viewing time and, as recommended [48], do not exceed 2 h of television per day, we found that they would be exposed to 4380 food and beverage commercials (including both core and non-core products) in a year, which would result in viewing 77 non-core products per week, and 4015 non-core products per year. Advertisements try to engage children with their messages, as they are both mediators with their parents and future consumers [49]. In this perspective, public health communication strategies should largely impact the socio-cultural environment, improving knowledge and the conscious adoption of behaviors that promote health [50,51]. The findings of our research might help future media literacy-based intervention. In actuality, educational campaigns aimed at teaching and communicating on consumer matters and media literacy are extremely needed [52]. The present study showed the entity of children’s exposure to food advertising and especially to those regarding unhealthy food and how persuasive techniques are used to drive children’s food choices. It should be taken into account that eating non-core food is an established risk factor for overweight and obesity. In this perspective, food advertising should be more strictly regulated, especially for commercials targeted to children. However, although there is evidence that TV food advertising contributes to the total intake of unhealthy foods, this evidence is mainly about short-term consumption. Data on long-term effects of food advertising exposure on children’s health are needed. Lastly, limiting TV view-time for children may be a useful application of the precautionary principle in order to reduce children’s exposure to food advertising.

### Strengths and Limitations of the Study

The main strength of this study is that a large sample of advertisements was recorded and assessed. Secondly, non-consecutive week and weekend days were included in the analysis. Furthermore, WHO protocol was used, highly impacting on the methodological quality of the study, as well allowing for a comparative study with other research studies that might be conducted in other countries in the future. Lastly, a wide set of variables was assessed. This monitoring work is fundamental in order to evaluate the efficacy of interventions aimed at improving the nutritional quality of food advertisements targeted to children. However, this study also has some limitations. Firstly, we only assessed Italian TV programs, and comparison with other countries is not possible at this time. Moreover, it was a cross-sectional analysis based on recording TV programs without collecting individual data on body mass index (BMI) or food choices. In this perspective, it was not possible to assess the impact of these food and beverage advertisements on children’s health outcomes. Lastly, we voluntarily and anonymously enrolled 33 parents. In spite of the apparent small sample size, it should be considered that the survey referred to a sample of 57 children, increasing the representativeness of the sample. Moreover, the results of our survey are consistent with previous research studies, thus confirming the validity of our study [53,54]. Nevertheless, our analyses refer to very large sample, since our results are based on 320 h of broadcasting TV for a total of 9069 commercials.

## 5. Conclusions

Children usually watch TV programs that transmit a high rate of food commercials, mainly advertising unhealthy products with captivating techniques. Food marketing was shown to play a key role in children’s food choices, and therefore it can contribute to the spread of childhood obesity. Legislators should advocate for healthy food marketing, trying to reduce both the exposure of children and the persuasive techniques that food brands can use to convince children to buy their products.

## Figures and Tables

**Table 1:** Prevalence of food and beverage categories.

	All Beverage and Food Products		Targeted to Youth	
Food Category	Total Products	Non-Core Products	Total Products	Non-Core Products
Total	1808 (100%)	1459 (80.7%)	610 (33.7%)	575 (31.8%)
Sweets	400 (22.1%)	400 (27.4%)	186 (30.5%)	186 (32.3%)
Composite and ready-made dishes	308 (17%)	296 (20.3%)	176 (28.9%)	176 (30.6%)
Bakery products	241 (13.3%)	241 (16.5%)	51 (8.4%)	51 (8.9%)
Other beverages	218 (12.1%)	128 (8.8%)	39 (6.4%)	34 (5.9%)
Breakfast cereals	75 (4.1%)	73 (5%)	55 (9%)	55 (9.6%)
Cheese	65 (3.6%)	29 (2%)	33 (5.4%)	4 (0.7%)
Yoghurts	59 (3.3%)	45 (3.1%)	24 (3.9%)	24 (4.2%)
Processed meat	59 (3.3%)	41 (2.8%)	1 (0.2%)	0
Milk drinks ^	48 (2.7%)	37 (2.5%)	0	0
Processed fruit and vegetables	44 (2.4%)	25 (1.7%)	0	0
Fresh frozen fruit and vegetables	42 (2.3%)	0	0	0
Fresh frozen meat	40 (2.2%)	0	0	0
Sauces	35 (1.9%)	17 (1.2%)	0	0
Fresh dry pasta	34 (1.9%)	0	0	0
Alcoholic beverages °	32 (1.8%)	32 (2.2%)	0	0
Edible ices *	31 (1.7%)	31 (2.1%)	28 (4.6%)	28 (4.9%)
Savory snacks	20 (1.1%)	20 (1.4%)	0	0
Juices	19 (1.1%)	19 (1.3%)	17 (2.8%)	17 (3%)
Fats and oils	18 (1%)	5 (0.3%)	0	0
Bread products	17 (0.9%)	17 (1.2%)	0	0
Energy drinks	3 (0.2%)	3 (0.2%)	0	0

^ Including milks and sweetened milks; almond, soya, rice and oat milks; but not cream. ° Including wine, beer, spirits, etc. * Including ice cream, frozen yoghurt, ice lollies, and sorbets.

## Supplementary Materials

The following are available online at https://www.mdpi.com/2304-8158/9/11/1632/s1: Table S1. Anonymous questionnaire administered. Table S2. Program category codes based on the WHO monitoring protocol and their definition [19].
