# Supplementary material for: Are the Italian Children Exposed to Advertisements of Nutritionally Appropriate Foods?
Source: Foods. 2020 Nov 8;9(11):1632. doi: 10.3390/foods9111632 (PMC7695182; doi:10.3390/foods9111632)
Supplement: Supplementary file 1 [file foods-09-01632-s001.pdf]

Table S1. Anonymous questionnaire administered.

Dear parent,

This questionnaire is part of a research project performed by the Perugia University. The aim of this project is to monitor advertisements aired by TV channels and directed to Italian children. For this reason we need to understand which TV channels are more viewed by children. This questionnaire is anonymous and no personal information will be collected. It is based on 4 simple questions and it will only take a few minutes. Your contribution is fundamental for our research. Thank you for your contribution.

1.How many children do you have in your family unit? *#

- ☐ 1 — *Skip to question 2*
- ☐ 2 — *Skip to question 5*
- ☐ 3 — *Skip to question 11*
- ☐ 4 — *Skip to question 20*

2.What is your first child's gender? *

- ☐ male
- ☐ female

3.How old is your first child? *

- ☐ from 1 to 5 years
- ☐ from 6 to 10 years
- ☐ from 11 to 13 years
- ☐ from 14 to 16 years
- ☐ Other_______________

4.Which of the following are the 5 most viewed channels by your first child. *

Tick all that apply.

- ☐ Boing
- ☐ K2
- ☐ Frisbee
- ☐ Rai yoyo
- ☐ Rai Gulp
- ☐ Cartoonito
- ☐ Rai 1
- ☐ Rai 2
- ☐ Rai 3
- ☐ Canale 5

- ☐ Italia 1
- ☐ Other______________

[#]In order to increase the number of answers to the questionnaire, parents had to provide information about the gender, age and the five most viewed channel for each of their children. The questionnaire was made so that depending on the number of children, parents were redirected to the section of the questionnaire where they could answer to the questions for each child.

* Required

Table S2. Program category codes based on the WHO monitoring protocol and their definition [19].

Programme	Progamme Definition
1= Comedy	Television series where humour was the main topic
2 = Drama	Television series where humour was not the main topic
3 = Movie	Programmes that where first aired in theatres
4 = Soap opera	Programmes with recurring characters dealing with daily events
5 = Music/Music video	Concerts and programmes where music was the main topic
6 = News/Commentary	Programmes that gave information or discussed actual events
7 = Talk show	Programmes with hosts talking about different topics
8 = Reality show	Characters being filmed on real-life situations
9 = Sports show	Sports competitions and programmes with sport as the main topic
10 = Entertainment/Variety show	Programmes with hosts doing different types of performances
11 = Documentary show	Programmes that gave a factual report on a given topic
12 = Game show	Programmes where participants played to win a prize
13 = Children's show	Programmes specifically targeted to children based on characters and themes of the programme
14 = Infomercial	Programmes made to advertise branded products
15 = Other	All programmes that did not fit the previous categories
